# Occurrence and Diversity of CRISPR Loci in *Lactobacillus casei* Group

**DOI:** 10.3389/fmicb.2020.00624

**Published:** 2020-04-08

**Authors:** Lan Yang, Weixun Li, Obaroakpo Joy Ujiroghene, Yang Yang, Jing Lu, Shuwen Zhang, Xiaoyang Pang, Jiaping Lv

**Affiliations:** Institute of Food Science and Technology, Chinese Academy of Agricultural Sciences, Beijing, China

**Keywords:** *Lactobacillus casei* group, CRISPR system, spacer, phage, genotyping

## Abstract

Clustered Regularly Interspaced Short Palindromic Repeats (CRISPR) is an adaptive immune system that resists foreign genes through nuclease targeting in bacteria and archaea. In this study, we analyzed 68 strains of *Lactobacillus casei* group from the NCBI GenBank database, and bioinformatic tools were used to investigate the occurrence and diversity of CRISPR system. The results showed that a total of 30 CRISPR loci were identified from 27 strains. Apart from three strains which contained double loci with distinguishable distributed sites, most strains contained only one CRISPR locus. The analysis of direct repeat (DR) sequences showed that all DR could form stable RNA secondary structures. The CRISPR spacers showed diversity, and their origin and evolution were revealed through the investigation of their spacer sequences. In addition, a large number of CRISPR spacers showed perfect homologies to phage and plasmid sequences. Collectively, our results would contribute to researches of resistance in *L. casei* group, and also provide a new vision on the diversity and evolution of CRISPR/Cas system.

## Introduction

Lactic acid bacteria are recognized as food safety grade microorganisms (Saad et al., 2013). They contribute to improve food nutrition and ameliorate food flavor. At the same time, they have various probiotic functions such as the regulation of intestinal flora (Corinna et al., 2016) as well as the improvement of immunity (Akoglu et al., 2015). *L. casei* group, a type of lactic acid bacteria, can transit strong acid environments in the stomach and be colonized in the intestinal mucosa, thus play a major role in the prevention and treatment of gastrointestinal diseases. *L. casei* group have been widely commercialized. Several related products occupy a huge market share, and they are favored and affirmed by consumers. They have also expanded from the initial field of conventional yogurt to health, medicine, vaccines and several other fields. However, phage contamination is still a very serious problem for the industry of lactic acid bacteria (Garneau and Moineau, 2011). Phages can lyse bacteria to influence their death, decrease viable counts, cause slow fermentation and even production failure. These detriments consequently result in the decline of their acid production, flavor and taste. Since phages can resist pasteurization, their occurrence is difficult to completely eliminate. They are capable of rapid spread and even destruction of an entire production chain, leading to huge economic losses. Thus, anti-phage ability of lactic acid bacteria is a potential problem that needs to be explored in order to solve the problem of their development for useful applications.

Bacteria had evolved a variety of strategies against phages, which includes CRISPR/Cas system (Rodolphe et al., 2007; Garneau et al., 2010). These strategies prevent infection by cutting and integrating genetic elements of foreign invaders. Bacteria are also immune to other foreign invaders with homologous sequences (John et al., 2009; Makarova et al., 2011; Stern et al., 2012). CRISPR/Cas systems are usually clustered together with short palindromic repeats (CRISPR) and CRISPR-related (Cas) genes (Alexander et al., 2005; Rodolphe et al., 2007). CRISPR arrays contain RNA coding sequences that target foreign elements. Cas proteins function as nuclease and helicase, with the ability to unravel and cut DNA double strands in order to cause double-strand breaks in certain cases (Poorna et al., 2007; Tautvydas et al., 2013). In the past decade, CRISPR systems had been discovered to include 2 classes, 6 types, and more than 20 subtypes (Makarova et al., 2017a, b). Subsequently, researchers had developed them as gene-editing tools (Martin et al., 2012; Burgess, 2013; Wenyan et al., 2013). Currently, CRISPR-Cas9 gene-editing technology is widely used in animals and plants. CRISPR/Cas systems also have enormous potential applications as additions in genetic editing. Recently, the approach of strain-typing based on CRISPR system had been widely used in microorganisms such as *Pseudomonas aeruginosa* (Alex et al., 2015), *salmonella enterica* (Pettengill et al., 2014), and *Helicobacter cinaedi* (Tomida et al., 2017). Among lactic acid bacteria, it is usually difficult to type closely related strains based on the 16s rRNA sequences. However, this problem could be solved using CRISPR genotyping. Cas proteins capture fragments from foreign genetic elements and insert them into CRISPR array after processing to create a new repeat- spacer unit. The events of these insertions usually occur at the leader end of CRISPR loci, and the earlier acquired sequence, which also called ancestral spacers, is at the other end. As the evolution progresses, the newly acquired spacers are arranged in turn, then the information of the spacers’ position can form an evolution timeline. Bacteria rarely share exactly same CRISPR system. Thus, more useful background information is provided with CRISPR genotyping than other methods of genotyping.

Bioinformatic analysis of CRISPR system in probiotics is crucial for the assessment of potential evolution in the prediction of immunity, it is also of great importance to food industry and other applications. Our study would contribute to providing useful information about the molecular mechanism of *L. casei* group against phage infection. In addition, it would lay a foundation for subsequent screening and breeding of commercial anti-phage-infected *Lactobacillus.*

## Materials and Methods

### CRISPR/Cas System Identification

The 68 *L. casei* group genomes obtained from the NCBI GenBank database^1^ were used to characterize CRISPR/Cas systems. Subsequently, the CRISPR loci of these genomes were identified by CRISPR-Cas++ webserver^2^, and the output option was set to default. In general, CRISPR-Cas++ webserver was used for the detection of “Questionable” and “Confirmed” CRISPR loci, which involved only little repeat-spacer sequence and carried additional Cas gene, respectively. Only “Confirmed” CRISPR loci were further researched for the diversity of CRISPR system.

### Repeat Structure Predict and Spacers Analyses

Similar repeat sequences were clustered and aligned by MEGA7.0 and DNAMAN 6.0 software (MEGA Inc., Memphis, TN, United States). RNA secondary structures were predicted by the RNA fold Web server^3^, using minimum free energy (MFE) with default parameters. The visual representation of CRISPR spacers were performed using CRISPRVIZ^4^, and each unique color combination represented one distinct spacer sequence.

### Protospacer Target

Protospacers were publicly identified against plasmid and phage genomic sequences using CRISPR target web server^5^. The protospacer with the most effective match was considered from the comparison between two sequences which showed below 3 mis-matches across the whole length of the spacer sequences. The spacer matches were then analyzed for hierarchical clustering using the Pheatmap package in R3.6.0 software.

## Results

### Identification of CRISPR-Cas Systems in *Lactobacillus casei* Group

A total of 68 *L. casei* group strains from NCBI GenBank Database were analyzed for the occurrence of CRISPR systems and included 11 *Lactobacillus casei* and 57 *Lactobacillus paracasei* (Table 1). Based on the CRISPR systems search results, a total of 30 confirmed CRISPRs which included Cas genes were identified among two investigated subspecies. The number of strains with confirmed CRISPR systems accounted for 39% of *L. casei* group and was close to the occurrence rate estimated at 40% for bacteria.

**TABLE 1:** CRISPR-cas systems in *L. casei* group.

Lactobacillus casei groups	Strain	Assembly	Type-subtype	CRISPR direction	No. spacer	Repeat sequence
Casei	BL23	GCA_000026485.1	TypeII-A	-	21	GTCTCAGGTAGATGTCGAATCAATCAGTTCAAGAGC
	12A	GCA_000309565.2	None			
	W56	GCA_000318035.1	TypeII-A	-	15	GTCTCAGGTAGATGTCGAATCAATCAGTTCAAGAGC
	ATCC 393	GCA_000829055.1	None			
	LC5	GCA_002192215.1	None			
	CECT 9104	GCA_900492555.1	TypeII-A	-	42	GTCTCAGGTAGATGTCGAATCAATCAGTTCAAGAGC
			TypeI-C	+	13	ATTTCAATTCACGCAGTCACGTAGACTGCGAC
			TypeI-C		25	ATTTCAATTCACGCAGTCACGTAGACTGCGAC
	A2-362	GCA_000510825.1	None			
	Z11	GCA_001885295.1	TypeII-A	+	11	GTTTTAGAAGGATGTTAAATCAATAAGGTTAAACCC
	MJA 12	GCA_002091975.1	None			
	YNF-5	GCA_004123005.1	None			
	DSM 20011	GCA_001433735.1	None			
Paracasei	ATCC 334	GCA_000014525.1	TypeI-E	-	20	GGATCACCCCCGCATGTGCGGGGAAAAC
	JCM 8130	GCA_000829035.1	None			
	Zhang	GCA_000019245.3	TypeII-A	-	16	GTCTCAGGTAGATGTCGAATCAATCAGTTCAAGAGC
	8700:2	GCA_000155515.2	TypeII-A	-	20	GTCTCAGGTAGATGTCGAATCAATCAGTTCAAGAGC
	BD-II	GCA_000194765.1	TypeII-A	-	21	GTCTCAGGTAGATGTCGAATCAATCAGTTCAAGAGC
	LC2W	GCA_000194785.1	TypeII-A	-	21	GTCTCAGGTAGATGTCGAATCAATCAGTTCAAGAGC
	LOCK919	GCA_000418515.1	TypeII-A	-	11	GTCTCAGGTAGATGTCGAATCAATCAGTTCAAGAGC
	N1115	GCA_000582665.1	None			
	CAUH35	GCA_001191565.1	None			
	L9	GCA_001244395.1	TypeII-A	-	46	GTCTCAGGTAGATGTCGAATCAATCAGTTCAAGAGC
	KL1	GCA_001514415.1	None			
	IIA	GCA_002079285.1	TypeII-A	-	35	GTCTCAGGTAGATGTCGAATCAATCAGTTCAAGAGC
	TK1501	GCA_002257625.1	TypeII-A	-	24	GTCTCAGGTAGATGTCGAATCAATCAGTTCAAGAGC
	FAM18149	GCA_002442835.1	none			
	TMW 1.1434	GCA_002813615.1	TypeI-E	-	130	GGATCACCCCCGCATGTGCGGGGAAAAC
			TypeII-A	-	22	GTCTCAGGTAGATGTCGAATCAATCAGTTCAAGAGC
	HD1.7	GCA_002865565.1	TypeII-A	-	32	GTCTCAGGTAGATGTCGAATCAATCAGTTCAAGAGC
	HDS-01	GCA_002902825.1	TypeII-A	-	30	GTCTCAGGTAGATGTCGAATCAATCAGTTCAAGAGC
	EG9	GCA_003177075.1	TypeII-A	-	17	GTCTCAGGTAGATGTCGAATCAATCAGTTCAAGAGC
	Lpc10	GCA_003199005.1	TypeII-A	+	22	GTCTCAGGTAGATGTCGAATCAATCAGTTCAAGAGC
	LC355	GCA_003268715.1	TypeII-A	-	52	GTCTCAGGTAGATGTCGAATCAATCAGTTCAAGAGC
			TypeI-E	+	65	GTTTTCCCCGCACATGCGGGGGTGATCC
	ZFM54	GCA_003627255.1	TypeII-A	+	18	GTCTCAGGTAGATGTCGAATCAATCAGTTCAAGAGC
	7112-2	GCA_003957435.1	TypeII-A	-	21	GTCTCAGGTAGATGTCGAATCAATCAGTTCAAGAGC
	IJH-SONE68	GCA_003966835.1	None			
	SRCM103299	GCA_004141835.1	None			
	LcY	GCA_000388095.2	TypeII-A	-	21	GTCTCAGGTAGATGTCGAATCAATCAGTTCAAGAGC
	ATCC 25302	GCA_000159495.1	None			
	KL1-Liu	GCA_000827145.1	None			
	1316.rep1_LPAR	GCA_001062665.1	None			
	1316.rep2_LPAR	GCA_001062695.1	None			
	844_LCAS	GCA_001066565.1	None			
	275_LPAR	GCA_001076595.1	None			
	525_LPAR	GCA_001076935.1	TypeII-A	-	48	GTCTCAGGTAGATGTCGAATCAATCAGTTCAAGAGC
	BM-LC14617	GCA_001636215.1	None			
	RI-210	GCA_001981715.1	None			
	RI-194	GCA_001982085.1	None			
	RI-195	GCA_001982095.1	None			
	CCC B1205		None			
	KMB_598	GCA_003367655.1	None			
	AM33-2AC	GCA_003434205.1	TypeI-E	-	31	GTTTTCCCCGCACATGCGGGGGTGATCC
	DTA83	GCA_003571925.1	TypeII-A	+	30	GTCTCAGGTAGATGTCGAATCAATCAGTTCAAGAGC
	UBLPC-35	GCA_003640765.1	None			
	FAM18108	GCA_003712245.1	None			
	FAM18110	GCA_003712265.1	None			
	FAM18105	GCA_003712275.1	None			
	FAM18123	GCA_003712325.1	None			
	FAM18119	GCA_003712385.1	None			
	FAM18113	GCA_003712395.1	None			
	FAM18149	GCA_003712485.1	None			
	FAM18133	GCA_003712525.1	None			
	FAM18172	GCA_003712585.1	None			
	FAM6012	GCA_003712745.1	None			
	FAM8374	GCA_003712825.1	None			
	FAM8407	GCA_003712835.1	None			
	FAM6165	GCA_003712875.1	None			
	FAM18126	GCA_003712925.1	None			
	FAM3248	GCA_003712935.1	TypeI-E	+	91	GTTTTCCCCGCACATGCGGGGGTGATCC
	LcA	GCA_000400585.1	TypeII-A	-	21	GTCTCAGGTAGATGTCGAATCAATCAGTTCAAGAGC
	DSM 20258	GCA_001436485.1	None			
						

**FIGURE 1:**
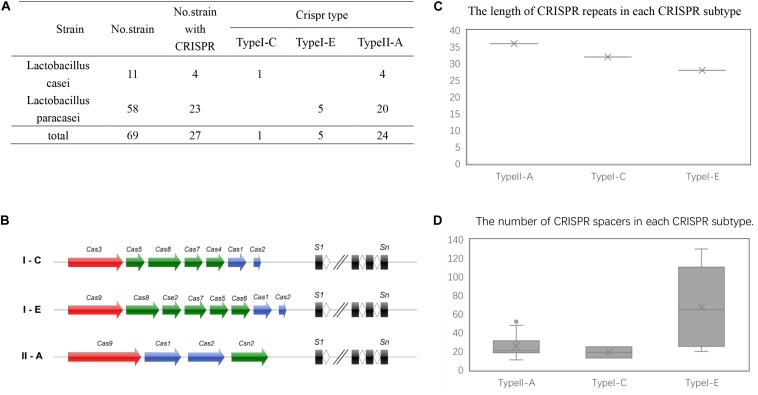
CRISP-Cas systems in *L. casei* group. **(A)** The number of CRISPR-Cas systems detected in *L. casei* group strains for each CRISPR-Cas type. **(B)** Schematic diagram of CRISPR-Cas systems in *L. casei* group. **(C)** The length of the CRISPR repeats in each subtype. The y-axis is the base number of CRISPR repeat sequences. **(D)** The number of CRISPR spacers for in the CRISPR loci of each subtype. The y-axis is the number of CRISPR spacers.

CRISPR/Cas system subtypes were confirmed by Cas genes species flanked by CRISPR arrays (Figures 1A,B). Among the strains with CRISPR loci, 24 *L. casei* group strains contained type II-A CRISPR locus, while 5 strains of the group contained type I-E CRISPR locus, only a single strain contained type I-C CRISPR locus. Interestingly, the type I-C locus had separate CRISPR arrays on both sides of Cas genes with similar repeat sequences, it was considered as the same. These confirmed CRISPR loci in the 30 strains were subsequently analyzed.

Repeat sequences were conserved in each CRISPR/Cas subtype. The length of the repeat sequences was 36 nucleotides for subtype II-A, 28 nucleotides for subtype I-E, and 32 nucleotides for I-C (Figure 1C). However, for subtype II-A CRISPR/Cas system, the number of spacers showed obvious polymorphisms, which ranged from 11 spacers in *L. paracasei* LOCK919 to 52 spacers in *L. paracasei* LC335. The variability in subtype I-E CRISPR/Cas system was high, from 20 spacers in *L. paracasei* ATCC334 to 130 spacers in *L. paracasei* TMW 1.1434. The unique subtype I-C system which was present in *L. paracasei* CECT 9104, contained 13 spacers on the upstream of Cas genes and 25 spacers on the downstream of Cas genes (Figure 1D).

### Diversity of Repeat and Spacer Sequences

**FIGURE 2:**
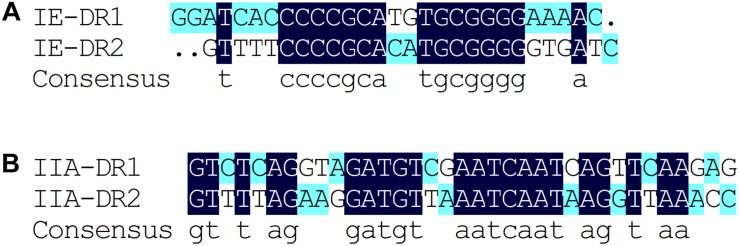
Alignment of type I-E **(A)** and type II-A **(B)** repeat sequences by DNAMAN, The dark blue represents completely identical sequences and the variation of nucleotide site is marked with other color.

**FIGURE 3:**
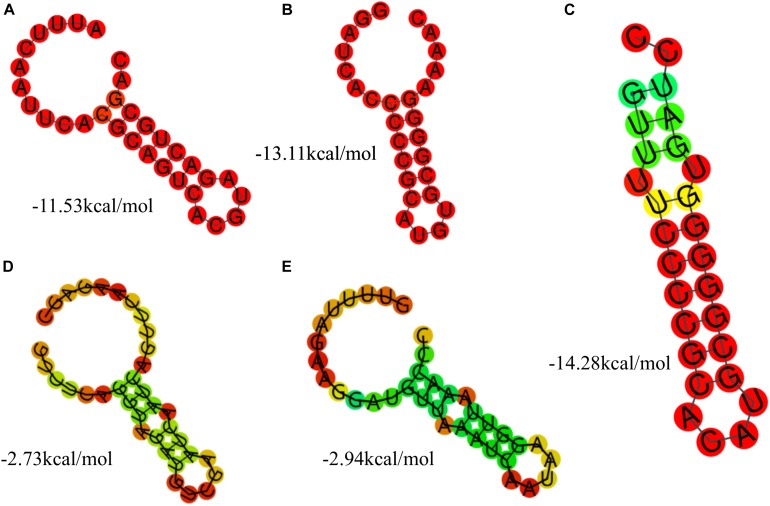
The prediction of DR secondary structure and MFE values in type I-C **(A)**, type I-E **(B,C),** and type II-A **(D,E)**.

Based on the results of the alignment of repeat sequences, 30 CRISPR loci in the 68 strains of *L. casei* group strains were divided into five groups, which included one group for subtype I-C, two groups for subtype I-E, and two groups for subtype II-A. The repeat sequences were conserved in the same subtype (Figure 2). The subtype II-A DR1 was the commonest sequence in 23 strains. In addition, the predicting results of DR structure showed that the RNA secondary structures formed stems in the middle. According to predictions, subtype I-C DR sequence included 7 bp stem length (Figure 3A), 7 and 10 bp for subtype I-E DR sequences (Figures 3B,C), 6 bp for subtype II-A DR sequence (Figures 3D,E).

The conservation and stability of the secondary structures of DR sequence could be analyzed from the diagram of the structure and MFE value. According to the algorithm of system optimization, the red graphic represented a high probability of formation, while the green graphic indicated that the structure had relatively low possibility. Overall, the stable RNA secondary structures tended to form long stems. In addition, the stability of the secondary structures could also be affected by the GC content. Repeats with higher GC content were more stable at the same stem length. In all groups, the secondary structures of DR in Subtype II system had mini MFE values and the formation of the shortest stem. However, the secondary structures of DR in Subtype I system had larger MFE comparable to those in Subtype II system although with similar stem lengths. This indicated that the GC content and the mismatched base numbers in the stem were in accordance with the stability of the secondary structure of RNA.

**FIGURE 4:**
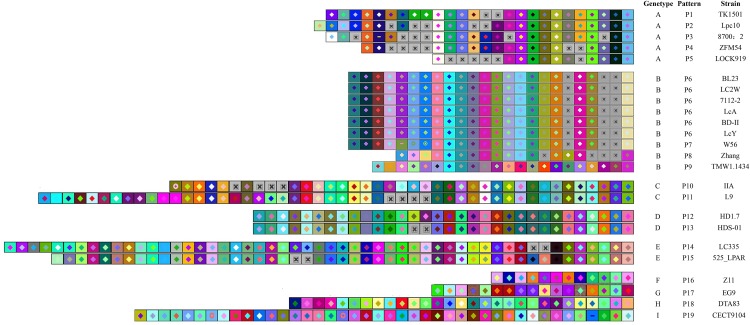
The type -II CRISPR arrays from *L. casei* group strains are represented graphically. The repeats have been eliminated and only spacers are shown. Identical spacers are shown as squares representing different color combination, Gray Squares containing an “X” represent no spacers. Strains are listed by CRISPR genotype, CRISPR array pattern, strain name. The newly acquired spacer is represented on the left side while the earliest acquired spacer is on the right side.

**FIGURE 5:**
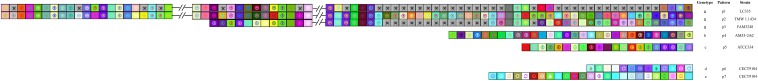
CRISPR subtype I spacers comparison in *L. casei* group. each unique spacer sequence is showed as a unique color combination. Gray Squares containing an “X” represent no spacers. Spacer is displayed from the ancestral end (right) toward the recently acquired spacers (left) in order.

The CRISPR spacers were analyzed to clarify the similarity and divergence of strains evolution under selective pressure from invasive DNA. As shown in Figures 4, 5, the strains could be grouped through the composition and distribution of spacers. The spacers were arranged from the ancestral (right) end toward the most newly acquired end (left), and each color combination represented a unique spacer sequence based on the nucleotide sequences. The strains with similar spacers were considered as one group, due to the fact that they could likely be initially exposed to the same environment. According to the spacer alignment, 9 type-II CRISPR genotypes were found which included 19 unique patterns (Figure 4). In addition, five different type-I CRISPR genotypes were found and included seven unique patterns (Figure 5). While BL23, LC2W, 7112-2, LcA, BD-II, and LcY completely shared identical spacers, other strains in the same group had different later acquired spacers. It is well known that same ancestral spacer may indicate a common origin, thus the acquisition of subsequent spacers could reflect different evolution. As a consequence, bacteria could be classified according to spacers.

### Spacers Homology to Phage and Plasmid

**FIGURE 6:**
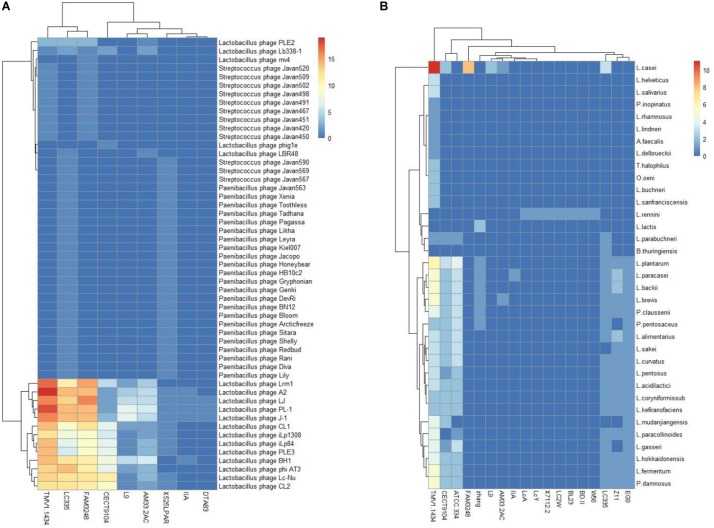
*L. casei* group CRISPR spacers targeting phages **(A)** and plasmid **(B)**. The heatmap represents the number of CRISPR spacers that matched phages **(A)** and plasmid **(B)**. The horizontal axis represents the strain that target phages **(A)** or plasmids **(B)**. The vertical axis represent phages **(A)** or plasmids **(B)** targeted by *L. casei* CRISPR spacers. The color scales represents the number of targeting events with blue squares representing the absent of matches and red squares representing the highest number of targeting.

The investigation of the similarity between CRISPR spacers and foreign DNA elements could be conducive for the unfold of immune information of strains, extraction of records of threats challenges encountered, as well as the rout of invasive DNA. Among the 27 *L. casei* group strains which harbored CRISPR/Cas systems, nine strains harbored at least one spacer targeting phages, while 18 strains displayed at least one spacer targeting plasmids (Figure 6). Interestingly, the *L. casei* group strains with type-I CRISPR systems presented more spacer targeting foreign DNA. The CRISPR/Cas system of *L. casei* TMV1.1434 harbored up to 11 spacers to target plasmids from *L. casei*. The most frequently match events happened with the plasmid sequences from *L. plantarum*, which was consistent with target of total plasmid. The reason could be as a result of the much largest number of sequenced plasmids from *L. plantarum* in the database. Moreover, 9*L. casei* group strains presented spacers targeting phage, the spacers of *L. casei* TMV1.1434 could match the maximum number of phages sequence, while half of *L. casei* CECT9104 spacers could match the phages. Regarding the diversity of species of matched spacers, *L. paracasei* LC335, *L. paracasei* 525 LPAR, and *L. casei* TMV1.1434 targeted up to 44, 36, and 24 different species of phages respectively.

PL-1 was the most frequently targeted phage. Also at the top of targeting were *L. casei* phage A2, phiAT3 and J-1. Notably, we analyzed the gene-targeted characteristics in phage PL-1, and some spacers shared a region of homology with the gene encoding tail component which played vital roles in phage replication. Similarly, multiple spacers matched the gene regions encoding major capsid protein or DNA packaging machinery. Thus, the immunity of CRISPR/Cas system could prevent phage replication via the destruction of these critical components, and enhance the viability of bacteria in phage-rich environment.

## Discussion

CRISPR system could resist foreign phages and plasmids through the mechanism of target interference by specific protein and guide RNA, thereby endue the bacteria strong adaptability to fight against complex environments (Makarova et al., 2013). Studies have found that about 40% of bacterial genomes contained CRISPR locus (Lillestøl et al., 2006), most CRISPR loci were located on their chromosomes and rarely on plasmids. The main reason for this phenomenon is that it will damage bacterial immunity hereditary if CRISPR-containing plasmids were lost. In the present study, we analyzed 68 strains of *L. casei* group, among which an extensive diversity was shown by CRISPR/Cas systems. Their different subtypes were harbored and included subtype IC, IE and IIA. It was obvious among different species there were diverse characteristics of CRISPR loci, thus provided a novel method of bacterial genotyping.

In general, direct repeat sequences of different CRISPR loci may exist differences. But they were conserved in same subtypes. Due to the presence of direct repeats of short palindromic sequences, double-stranded RNA secondary structures can be transcripted from the CRISPR array and combine with Cas proteins to target sites (Alexander et al., 2005; Tautvydas et al., 2013). The stem-loop structure of direct repeats may contribute to the interaction between RNA and Cas protein. As a consequence, the function of CRISPR loci may be affected by the stability of RNA secondary structures. Interestingly, in our study, the secondary structures of same subtypes were conserved, with similarities in their structural composition and free energy, irrespective of their differences in repeat sequences. It suggested that the repeat sequences were diverse in the process of evolution, although the function could be conserved. In addition, according to MFE value theory, longer match base numbers and higher GC contents in stem tended to form stable secondary structures, and secondary structures of RNA with lower minimum free energies were more stable. In understanding the evolution of bacteria, the genotyping analysis was crucial although excessive data analysis and high cost of sequencing had mainly hindered its widespread use. Another genotyping method commonly used is multilocus sequence typing (MLST), which is based on the nucleotide sequences of seven housekeeping genes. Tomida et al. (2017) determined a genotyping method based on CRISPR spacer and compared it with the methods of MLST genotyping using 42 *H. cinaedi* strains, the results showed MLST had little variability while the CRISPR spacer sequences showed remarkable diversity (Tomida et al., 2017). Morovic et al. (2016) showed that 42% of the commercial dietary supplements contained incorrectly labeled microorganism regarding taxonomy. Lewis et al. (2016) showed that 15 of 16 commercial probiotics in this study products present bacterial compositions that differed from the list of ingredients. Thus, the accessorial genotyping and correct identification methods were seriously needed as additions to traditional tools. The CRISPR/Cas systems had been used for the identification of various pathogens. However, rare reports are available about the application of genotyping to probiotics via CRISPR systems. Riedel et al. analyzed CRISPR systems and genotyped strains via spacer sequence in *Bifidobacterium* (Riedel et al., 2015; Hidalgo-Cantabrana et al., 2017), thereby created an awareness about the potential of the CRISPR system in probiotic genotyping. In this study, we considered CRISPR spacers as genotyping tools in *L. casei* group, in order to distinguish closely related strains. Different strains were specifically distinct, the later acquired spacers were diversified despite that they shared the same ancestral spacer. Only a few strains shared exact same spacers. Similar to other methods, the CRISPR/Cas genotyping method was largely limited and could be attributed to the absence of CRISPR systems in some strains. Therefore, the combination of multiple genotyping methods could be a developmental trend in the future. CRISPR spacers represent the immunity records of strains suffering from invasive DNA. The results of our study showed that less than half of spacers could match phages or plasmids. Only CECT9104 reached half of the spacers, while most of the other strains had only one or none. The limited number of spacer matches could be attributed to the presence of substantial plasmids or phages that had not been sequenced, or constantly evolve lead to escape mechanism. Strains obtained an evolutionary advantage from CRISPR-Cas systems by recording immune information, thereby prevented DNA invasion again. The *L. casei* group harboring CRISPR/Cas immune systems would be suitable as industrial probiotics against viral challenges. They could also have the potential to fight abundant phages in the gut.

## Conclusion

In conclusion, our findings confirmed that *L. casei* group strains harbored diverse CRISPR/Cas systems. Furthermore, the results of the bioinformatic analysis could provide a data basis for broader CRISPR studies in *L. casei* group. The polymorphism of CRISPR system showed its potential for the genotyping of strains as well as the immunity of strains against invasive DNA. The CRISPR/Cas system analysis of *L. casei* group provided new insights into the diverse roles of CRISPR/Cas system in probiotic in this study.

## Data Availability Statement

The datasets generated for this study can be found in the https://www.ncbi.nlm.nih.gov/genome/genomes/652, https://www.ncbi.nlm.nih.gov/genome/genomes/2032.

## Author Contributions

LY, WL, YY, and XP collected the data and performed experiments. JL, SZ, XP, and JPL designed the study. LY and WL wrote the original draft. OU made multiple revisions and edited the manuscript. All authors contributed to the writing, reviewing, and editing of the manuscript. All authors read and approved the final version of the manuscript.

## Conflict of Interest

The authors declare that the research was conducted in the absence of any commercial or financial relationships that could be construed as a potential conflict of interest.

## Footnotes

1 https://www.ncbi.nlm.nih.gov/genbank/

2 https://crisprcas.i2bc.paris-saclay.fr/CrisprCasFinder/Index

3 http://rna.tbi.univie.ac.at/cgi-bin/RNAWebSuite/RNAfold.cgi

4 https://github.com/CRISPRlab/CRISPRviz

5 http://crispr.otago.ac.nz/CRISPRTarget/crispr_analysis.html
